# COVID-19 and informal settlements: an urgent call to rethink urban governance

**DOI:** 10.1186/s12939-020-01198-0

**Published:** 2020-06-03

**Authors:** S. Van Belle, C. Affun-Adegbulu, W. Soors, Prashanth N. Srinivas, G. Hegel, W. Van Damme, D. Saluja, I. Abejirinde, E. Wouters, C. Masquillier, H. Tabana, F. Chenge, K. Polman, B. Marchal

**Affiliations:** 1 Department of Public Health, Institute of Tropical Medicine, Antwerp, Nationalestraat 155, 2000 Antwerp, Belgium; 2 Institute of Public Health, Bangalore, India; 3 INCAP Research Center for the Prevention of Chronic Diseases (CIIPEC), Institute of Nutrition of Central America and Panama (INCAP), Guatemala City, Guatemala; 4 Independent Consultant, New Delhi, India; 5 Centre for Global Child Health, Hospital for Sick Children, Toronto, Canada and Dalla Lana School of Public Health, University of Toronto, Toronto, Canada; 6 Centre for Population, Family & Health, University of Antwerp (Belgium) and Centre for Health Systems Research & Development, University of the Free State (South Africa), Sint - Jacobstraat 2 -4, 2000 Antwerp, Belgium; 7 Centre for Population, Family & Health, University of Antwerp (Belgium), Sint - Jacobstraat 2 -4, 2000 Antwerp, Belgium; 8 School of Public Health, University of the Western Cape, Cape Town, South Africa; 9 School of Public Health, University of Lubumbashi and Health Knowledge Centre of the Democratic Republic of Congo, Lubumbashi, Democratic Republic of Congo; 10 Department of Biomedical Sciences, Institute of Tropical Medicine, Antwerp, Nationalestraat 155, 2000 Antwerp, Belgium

While some countries are nearing or reaching their peak of coronavirus infections, others are only at what seems to be the early stages of the infection curve. Some of these countries, particularly in the Global South, contain some of the world’s largest informal and/or urban settlements and are low resource settings. Given that the last few months have shown us how quickly COVID-19 can push health systems to the brink or overwhelm them, even in high-income countries, it is worrying to think what would happen if the outbreak becomes severe in such contexts.

The question is, how can outbreaks of COVID-19 in informal settlements in the South be prevented from triggering even wider shocks? Informal settlements, in addition to making up not a substantial proportion of urban populations, also present all the conditions for rapid spread: very high population density, scant access to water and sanitation, widespread poverty and inadequate health infrastructure. Indeed, favelas, barrios, slums and shantytowns seem to be the Achilles heel of many health systems, yet, political leaders in low- and middle-income countries have largely been silent about how they plan to face this significant but extremely important challenge. This may due to the uncertainty surrounding almost every aspects of the virus as well as the difficulties associated with defining and implementing an effective response. However, it is not inconceivable that this silence is the result of the contentious relationship between city authorities and people living in informal settlements.

Spurred by urban planning discourse originating in the US and the UK and real estate development logics, many city authorities have long adopted slum eradication policies and de facto ignored slum dwellers. The result is often political neglect and social and political exclusion [1], which contribute to distrust and sometimes outright fear of the authorities. In many cities, residents of informal settlements and government are locked in permanent conflict, which is rooted in histories of structural violence and social injustice [1]. In South Africa, for instance, shack settlements are sites of defiance, as vulnerable communities feel excluded from the political process. Last month, grassroot organisations in Khayelitsha, Cape Town’s largest township, protested to have water delivered by the city authorities to combat COVID-19 [2].

The recent Ebola epidemics in West Africa and DRC provide other examples of how distrust affects the response to epidemics. In Eastern DRC, distrusting communities slowed down the battle against Ebola [3], and when the police tried to impose a quarantine in the West Point slum of Monrovia, violence erupted [4]. Effective urban governance and trust are at the center of effective outbreak control, yet they are, by definition bound to be, absent in informal settlements.

Two consequences of the absence of public services and formal governance in informal settlements need to be considered. First, inadequate public services render their residents even more vulnerable. In many cases, residents need to negotiate access to scant public services through ‘middlemen’ who operate clientelistic networks, including ‘slumlords’ and local government authorities [5]. In such conditions, service provision becomes an instrument of exploitation of vulnerable people with, for instance, basic public services being effectively privatized. Examples can be found in informal settlements in Kenya, Ghana and India where operators charge exorbitant prices for access to toilets or drinkable water [6]. Such ‘actual’ service delivery excludes the most vulnerable groups.

Second, the absence of formal governance in informal settlements does not equal a lack of governance. Non-state organisations or resident-led initiatives tend to step in, often responding to basic needs and in the process setting up a bottom-up, networked governance system [7]. Church leaders, for instance, are often community liaisons and powerful brokers when slum dwellers distrust government intervention - in Ghana, traditional leaders disbanded all mass gatherings and funerals in the light of the pandemic. The governance vacuum is sometimes filled by what (maybe too easily) is referred to as ‘gangs’. These gangs may become actors in slum governance, as in South Africa’s townships or Brazil’s favelas, where for instance, gangs in the Cidade de Deus favela in Rio de Janeiro imposed a curfew [8]. Whatever the nature of the governance arrangements that emerge in the absence of formal governance structures, the resulting ‘real governance’ [9] needs to be understood in order to develop and implement effective measures. As the Ebola outbreaks taught us, experts only became effective once they started listening to local communities [10]. Yet, while real governance arrangements arguably fill gaps, they may lack accountability: who represents who in these arrangements and with which legitimacy?

The COVID-19 pandemic is a wake-up call for city authorities to rethink their engagement with the people living in informal settlements. It highlights once again how governance, health and equity are intertwined, and demonstrates the fact that effective urban governance cannot be achieved without collaboration with and/or the engagement of residents and real governance actors. Neglecting public services and accountability in informal settlements and ignoring the insights of the real governors will be counterproductive both in the control of the current pandemic and the prevention and management of future epidemics.

## Data Availability

Not applicable.
